# Loosening and revision rates after total shoulder arthroplasty: a systematic review of cemented all-polyethylene glenoid and three modern designs of metal-backed glenoid

**DOI:** 10.1186/s12891-020-3135-6

**Published:** 2020-02-21

**Authors:** Dong Min Kim, Mohammed Aldeghaither, Fahad Alabdullatif, Myung Jin Shin, Erica Kholinne, Hyojune Kim, In-Ho Jeon, Kyoung-Hwan Koh

**Affiliations:** 1 Department of Orthopaedic Surgery, Asan Medical Center, University of Ulsan College of Medicine, 88 Olympic-ro 43-gil, Songpa-gu, Seoul, 05535 South Korea; 2 College of Medicine, King Saud University, Riyadh, Saudi Arabia; 3 Department of Orthopaedic Surgery, St Carolus Hospital, Jakarta, Indonesia

**Keywords:** Shoulder, Osteoarthritis, Arthroplasty, Glenoid component, Metal-back, Polyethylene

## Abstract

**Background:**

Several modern designs of metal-backed glenoids (MBG) have been devised to overcome flaws such as loosening and a high failure rate. This review aimed to compare rates of complications and revision surgeries between cemented polyethylene glenoid (PEG) and three examples of modern MBG designs.

**Methods:**

Literature search was carried out using PubMed, Cochrane Library, EMBASE, and Google Scholar using MeSH terms and natural keywords. A total of 1186 articles were screened. We descriptively analyzed numerical data between the groups and statistically analyzed the categorical data, such as the presence of radiolucent line, loosening, and revision surgery (failure). Articles were divided into three groups based on follow-up duration: < 36-month, 36–72-month, and > 72-month subgroups.

**Results:**

This study included 35 articles (3769 shoulders); 25 on cemented PEG and ten on the modern MBG. Mean age was 66.4 (21–93) and 66.5 years (31–88). The mean duration of follow-up was 73.1 (12–211) and 56.1 months (24–100). Overall, the rate of the radiolucent line was 354/1302 (27%) and 47/282 (17%), the loosening rate was 465/3185 (15%) and 22/449 (5%), and the failure rate was 189/3316 (6%) and 11/457 (2%), for PEG and MBG, respectively. The results of < 36-month and 36–72-month subgroups showed lower rates of radiolucency and loosening in the cemented PEG group, but there was no significant difference in failure rate (*P* = 0.754 and 0.829, respectively). In the > 72-month subgroup, MBG was better in terms of loosening (*P* < 0.001) and failure rates (*P* = 0.006).

**Conclusions:**

The modern MBG component, especially TM glenoid, seems to be a promising alternative to cemented PEGs, based on subgroup revision rates according to the follow-up duration and overall results of ROM and clinical scores. All polyethylene glenoids tend to increase loosening and failure over time. Three modern MBG designs seem to have no difference in failure, at least in the < 36-month and 36–72-month subgroups compared to the cemented PEG. More long-term follow-up studies on modern MBG should be ultimately conducted.

**Level of evidence:**

Level IV, systematic review.

## Background

Although numerous studies on total shoulder arthroplasty (TSA) have aimed to find the optimal TSA design, no definite conclusions have been made [1]. The glenoid component of TSA is divided into keel type and peg type according to its shape, and can be made of all polyethylene (PE) or be metal-backed. Both metal-backed glenoids (MBG) and cemented polyethylene glenoids (PEG) were initially used, however due to the nature of the initial MBG design, the polyethylene liner was very thin and resulted in a high wear and failure rate [2]. A systematic review conducted in 2014 concluded that MBGs are not recommended as they show higher failure rates [3].

However, advanced MBG designs were devised to address these shortcomings, increasing the chance of good clinical outcomes [4–6]. We aimed to summarize and compare the results of TSA using cemented PEG and modern MBG by examining radiolucency, loosening, and failure rate. Our null hypothesis was that radiolucency, loosening, and failure rates of modern MBGs would be similar to those of cemented PEG.

## Methods

This systematic review was conducted in accordance with the preferred reporting items for systematic reviews and meta-analyses (PRISMA) guidelines [7]. Additionally, we have registered the current review on the website of International prospective register of systematic reviews (PROSPERO, CRD42019137134).

### Inclusion and exclusion criteria

We regulated various factors that could cause heterogeneity using strict inclusion and exclusion criteria determined by group discussion. Articles eligible for inclusion had to be a study on adults (> 18 years old), be a clinical study presenting the results of TSA using the cemented PEG or modern MBG with more than a 2 year mean follow-up (FU), a study including any type of shoulder arthritis, and be written in English. Case reports or articles with fewer than 5 cases were excluded. Also, articles that show the results of hybrid cage glenoids, mixed cases of revision arthroplasty, or mixed cases of structural bone graft, and articles which do not present the main outcomes (number of revisions or failure) were excluded.

### Search strategy and study selection

PubMed, Embase, Google Scholar, and Cochrane Library were searched to find a large number of relevant articles. We conducted group discussions and consulted medical informatics experts for an effective search strategy. After such discussions and consultations, we decided to search for final articles using individual search terms for MBG and PEG, respectively. The search terms for articles on cemented PEG were “total AND shoulder AND (replacement OR arthroplasty) AND polyethylene”. Search terms for articles on modern MBG were “total AND shoulder AND (replacement OR arthroplasty) AND (metal OR backed OR (cementless glenoid))”. After excluding duplicated documents, two independent reviewers screened the title and abstract, and finally selected articles through full-text review. We also performed citation tracking and search updates to find additional related articles using Google Scholar as an additional tool. All disagreements were resolved through group discussions of three or more authors.

### Methodological assessment and data extraction

Levels of evidence were assessed according to the Oxford Center for Evidence Based Medicine [8]. The methodological quality of the studies included in this review was assessed using the methodological index for non-randomized studies (MINORS) [9]. A total of 8 items were evaluated for non-comparative studies, and 12 items for comparative studies. As 0, 1, or 2 points can be assigned to each item, non-comparative studies can have a total of 16 points, while comparative studies can have a total of 24 points. A study that obtained more than 60% of the total score was considered as a high-quality article, and the distribution of high-quality articles was analyzed between the two groups.

In order to define “modern design”, the core topic of this study, the most up to date articles on the glenoid component were reviewed in group discussion. The advanced MBG designs presented by Castagna and Garofalo, who comprehensively assessed the product development year, conformity, rod, keel shape, and material, were defined as modern MBGs [10]. We included three designs in the modern MBG group: 1) second-generation SMR MBG (SMR System, Lima Corporate, Villanova, di San Daniele, Udine, Italy), 2) first-generation trabecular metal (TM) glenoid which consists of a soft MBG, the Sulmesh (Zimmer, Winterthur, Switzerland), and 3) the second-generation TM glenoid (Zimmer, Winterthur, Switzerland). If studies on the recent MBG design (after 2010) which was not one of the three designs mentioned above were found, we decided to conduct a group discussion. No such study was found, so the three designs were finally considered “modern design”.

Three independent reviewers extracted the number of shoulders, age, sex, FU duration, surgery procedures, medical and surgical history, preoperative diagnosis, name of implant and manufacturer, clinical score, range of motion (ROM), radiologic FU such as radiolucent lines, loosening, other complications, and revision or failure from the articles. The radiolucent line was defined as a radiolucency of 1 mm or more, grade 2 or more on the Lazarus radiolucency scoring system, or seven or more points out of a total of 18 points [11]. Failure was defined as complications that resulted in revision surgery involving an implant-related procedure. Loosening included both radiological and clinical loosening. Data presented by other methods and ambiguous data were not extracted.

### Statistical analyses

We used strict criteria to minimize heterogeneity. However, trends in age, FU duration, and preoperative diagnosis could be identified after data extraction. In particular, FU duration was considered to be the most important variable associated with implant failure. We collaborated with medical statisticians on data interpretation and data analysis (including scatter plot and subgroup analysis). For categorical variables such as the presence of radiolucent lines, loosening, and failure or revision surgeries, statistical analysis was performed on the difference between cemented PEG and modern MBG.

Since the FU duration varies from study to study, we determined that a simple overall comparison between 2 groups was not sufficient, and therefore two additional analyses were performed according to the FU duration. Firstly, a scatter plot was used that plots the mean FU duration and loosening and revision rates of each study. Trend lines were weighted according to the number of cases to identify trends of loosening and revision rates between the two groups. Secondly, a subgroup analysis was performed that divided the FU duration into three groups based on 36 and 72 months as the statistician suggested. Articles were divided into 3 groups based on follow-up duration: < 36-month, 36–72-month, and > 72-month subgroups. Subsequently, we analyzed the radiolucency, loosening, and revision rates overall, and for the < 36-month, 36–72-month, and > 72-month subgroups. All statistical analyses were performed using R version 3.5.1 (R Foundation for Statistical Computing, Vienna, Austria). *P*-values less than 0.05 were determined to be statistically significant. Since numerical data were often missing important values such as standard deviation, a meta-analysis could not be performed. Therefore, descriptive analysis and weighted means were performed on the numerical data of 2 groups.

## Results

### Search results

**Fig. 1:**
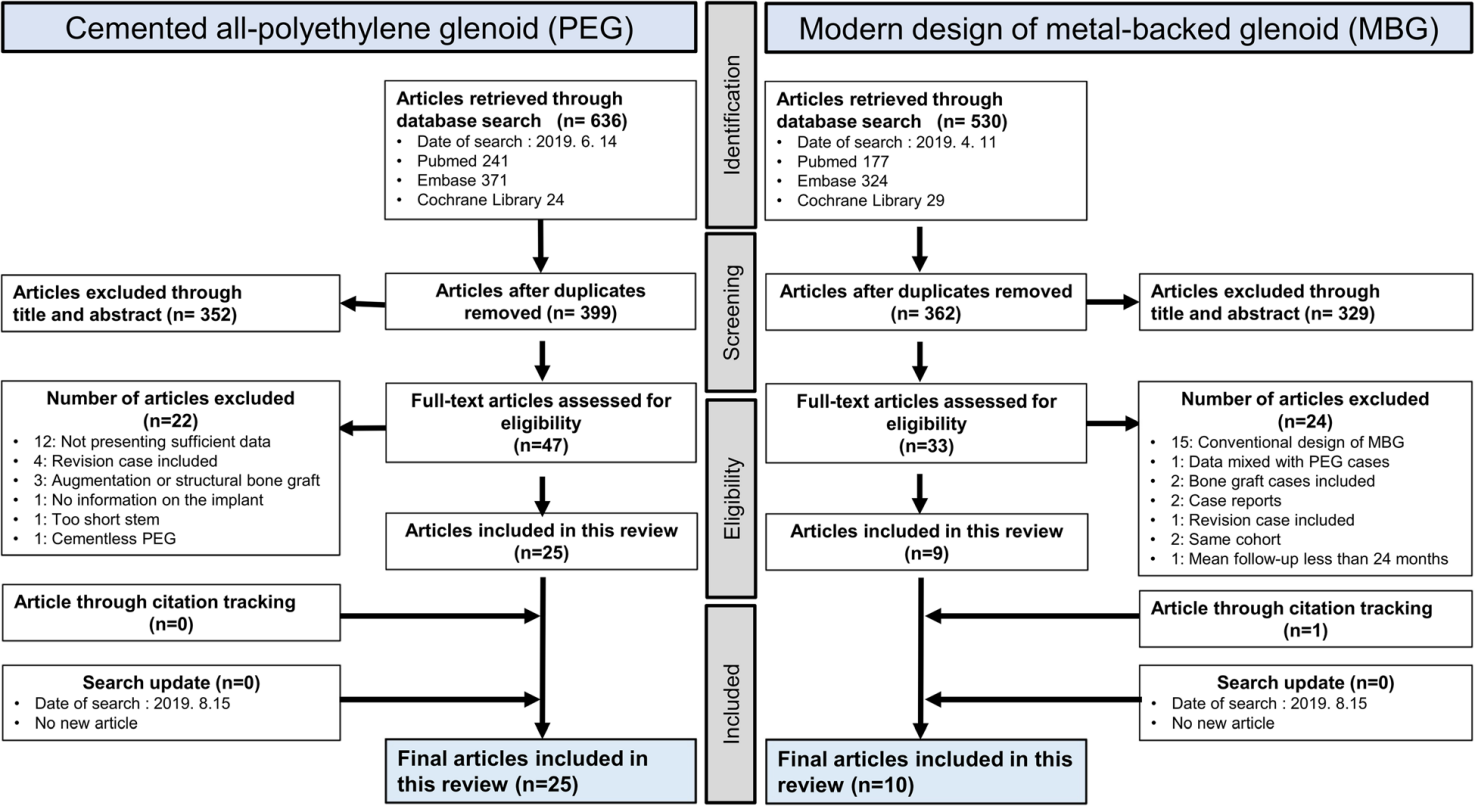
PRISMA flow diagram showing the selection of appropriate articles

Two hundred forty-one articles on cemented PEG were found in PubMed, 371 in Embase, and 24 articles in Cochrane Library. Subsequently, 177 articles on modern MBG were found in PubMed, 324 articles in Embase, and 29 articles in Cochrane Library. Through screening titles and abstracts and using full-text review, 25 PEG and 9 MBG articles were included. One article was added through citation tracking of selected articles, and no additional articles were found in the search update (Fig. 1). The final cemented PEG group included 3312 patients (25 articles) [12–36], and the modern MBG group included 457 patients (10 articles) [4–6, 37–43].

### Assessment of methodological quality and heterogeneity between two groups

**Fig. 2:**
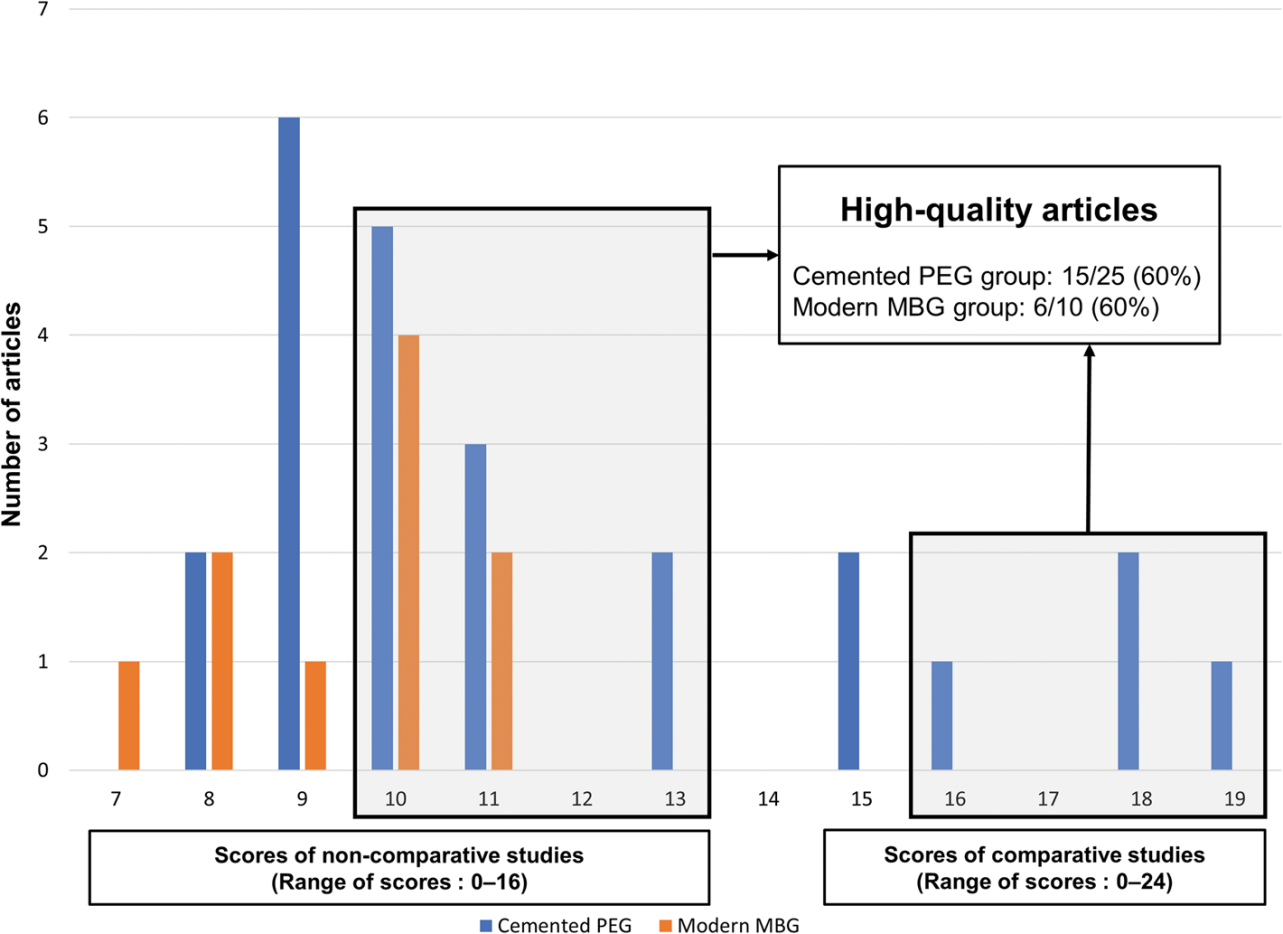
“Methodological index for non-randomized studies” scores of individual articles and the range that indicates high-quality articles. PEG, polyethylene glenoid; MBG, metal-backed glenoid

Levels of evidence and MINORS scores were determined by agreement between the two investigators, and there was no disagreement; one randomized controlled trial (Level I), one prospective comparative study (Level II), five Level III studies, and 28 Level IV studies were included. The mean MINORS scores, except for one Level I study, were 9.75 ± 1.38 for non-comparative studies and 16.8 ± 1.57 for comparative studies. Fifteen of the 25 studies on the cemented PEG (including Level I study, 60%) and 6 of the ten studies on the modern MBG (60%) were classified as high-quality articles (Fig. 2).

**Fig. 3:**
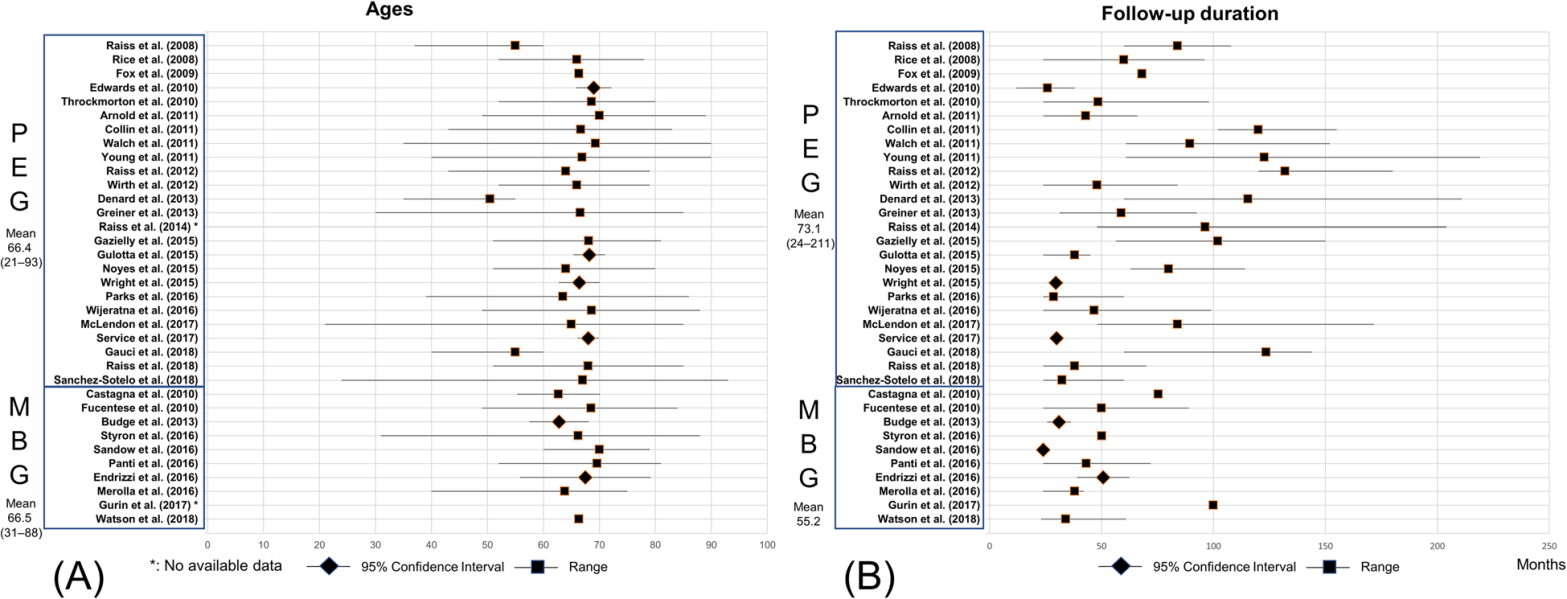
(**a**) Summary plots for age (**b**) Summary plots for follow-up duration. PEG, polyethylene glenoid; MBG, metal-backed glenoid

**Fig. 4:**
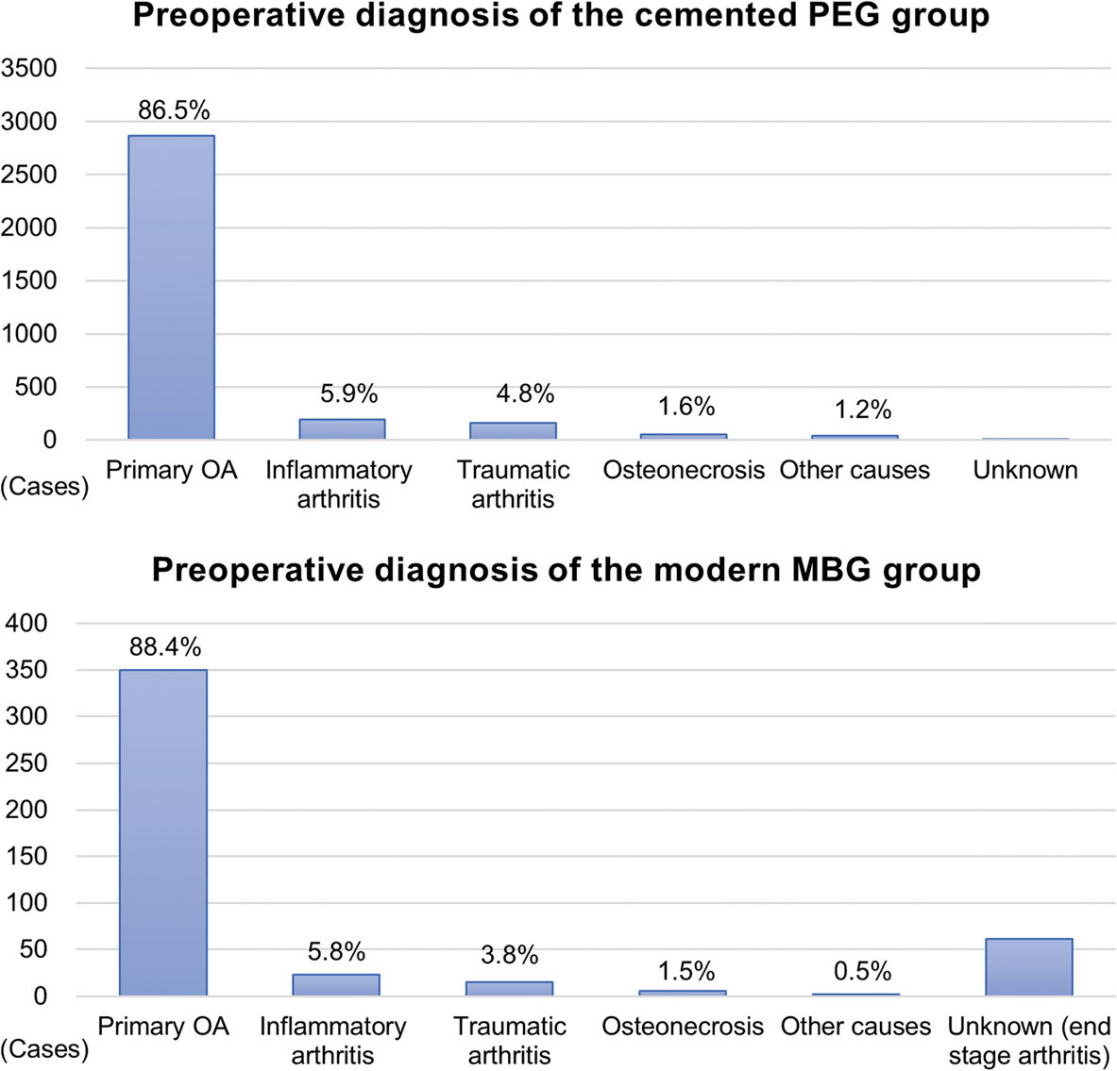
Graph showing the distribution of preoperative diagnosis for each group. PEG, polyethylene glenoid; MBG, metal-backed glenoid; OA, osteoarthritis

We analyzed the distribution of three factors that could introduce heterogeneity. Age and FU duration are shown using the summary plot (Fig. 3a and b). Age showed a similar pattern except for three studies in the PEG group with young adults, whereas the cemented PEG group tended to have a longer FU period than the modern MBG group. The distribution of preoperative diagnosis was similar between the two groups (Fig. 4), and the proportion of primary osteoarthritis was not statistically different (*P* = 0.310).

### Summary of outcomes of each article

**Fig. 5:**
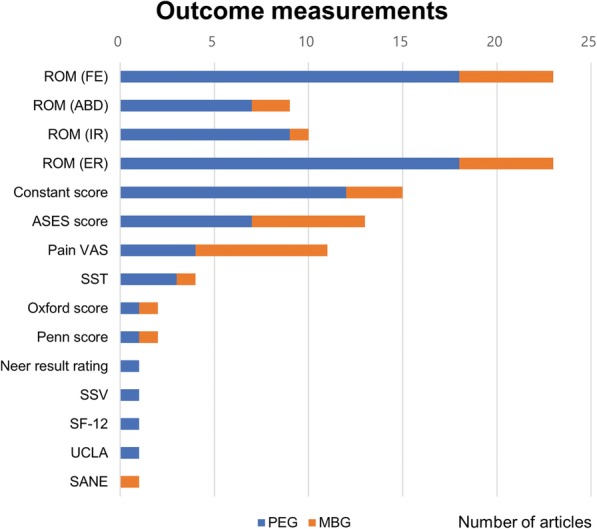
Distribution of outcome measurements. FE, forward elevation; ABD, abduction; IR, internal rotation; ER, external rotation; ASES, American shoulder and elbow surgeons; VAS, visual analogue scale; SST, simple shoulder test; SSV, subjective shoulder value; SF-12, short form-12; UCLA, University of California at Los Angeles; SANE, single alpha-numeric evaluation

Table 1 shows the demographic data and the outcome measurements of each study. Each study used a variety of measures; commonly used items were forward elevation (FE, 18 and 5 articles for cemented PEG and modern MBG, respectively), external rotation (ER, 18 and 5 articles), Constant score (13 and 3 articles), and ASES scores (7 and 6 articles), pain visual analogue scale (VAS, 5 and 7 articles), complications (most articles), and revision surgeries or failure (all articles) (Fig. 5). The results for each article for each commonly used item are shown in Table 2.

**Table 1:** Demographic data and outcome measurement of individual studies

Authors	Level of Evidence	Design	Cases	Mean age (y, range)	Mean FU (m, range)	Range of motion	Outcome measurements
Cemented all-polyethylene glenoid components (PEG)							
Raiss (2008)	IV	Aequalis^a^	21	55 (37–60)	7 years (5–9)	FE, ABD, IR, ER	Constant score
Rice (2008)	IV	Cofield II^b^	14	66 (52–78)	5 years (2–8)	ABD, ER	Neer result rating
Fox (2009)	IV	Mixed^c^	972	66.4	68.1	N	N
Edwards (2010)	I	Aequalis^a^	47	69 ± 11	26 (12–38)	N	N
Throckmorton (2010)	III	Cofield	100	68.6 (52–80)	48.5 (24–98)	FE, IR, ER	VAS
Arnold (2011)	IV	Global Advantage^d^	35	70 (49–89)	43 (24–66)	N	Constant score
Collin (2011)	II	Aequalis^a^	56	66.7 (43–83)	120 (102–155)	N	N
Walch (2011)	IV	Aequalis^a^	333	69.3 (35–90)	89.5 (61–152)	FE, ER	Constant score
Young (2011)	IV	Aequalis^a^	226	66.9 (40–90)	122.7 (61–219)	FE, ER	Constant score
Raiss (2012)	IV	Aequalis^a^	39	64 (43–79)	132 (120–180)	FE, ABD, IR, ER	Constant score
Wirth (2012)	IV	Global Advantage^d^	44	66 (52–79)	48 (24–84)	FE, IR, ER	VAS, ASES score, SST
Denard (2013)	IV	Aequalis^a^	50	50.5 (35–55)	115.5 (60–211)	FE, ER	Constant score
Greiner (2013)	IV	Affinis^e^	97	66.6 (30–85)	58.8 (31.2–92.5)	FE, ABD	Constant score
Raiss (2014)	IV	N (mixed)	329	N	8.0 years (4–17)	FE, ER	Constant score
Gazielly (2015)	IV	Aequalis^a^	39	68.1 (51–81)	102 (56.4–150)	FE, ER	Constant score, pain score
Gulotta (2015)	III	BioModular^f^	40	68.2 ± 9.1	38 (24–45)	N	VAS, ASES
Noyes (2015)	IV	Global Advantage^d^	42	64 (51–80)	80 (63–114)	FE, ER	ASES
Wright (2015)	IV	Equinoxe^g^	24	66.4 ± 9.1	29.6 ± 8.7	FE, ABD, IR, ER	Constant score, ASES, SST, UCLA
Parks (2016)	IV	Affiniti^h^	76	63.5 (39–86)	28.7 (24–60)	FE, ABD, IR, ER	Constant score, ASES
Wijeratna (2016)	IV	Global Advantage^d^	83	68.6 (49–88)	46.7 (24–99)	FE, IR, ER	ASES, Oxford score
McLendon (2017)	IV	Cofield II^i^	287	65 (21–85)	84 (48–171.6)	N	N
Service (2017)	III	Global Advantage^d^	71	68 ± 8.3	30 ± 7.2	N	SST
Gauci (2018)	III	Aequalis^a^	46	55(40–60)	123.6 ± 26 (60–144)	FE, ER	VAS, Constant score, SSV
Raiss (2018)	IV	Aequalis^j^	118	68 (51–85)	38 (24–70)	N	N
Sanchez-Sotelo (2018)	2018/IV	PEG	202	67 (24–93)	32.4 (24–60)	FE, IR, ER	ASES
Modern design of metal-backed glenoid component (MBG)							
Castagna (2010)	IV	Second-generation SMR^l^	35	62.7 (55.3–70.1)	75.4	N	VAS, Constant score, SST
Fucentese (2010)	IV	Sulmesh^m^	22	68.5 (49–84)	50 (24–89)	N	Constant score
Budge (2013)	IV	Tantalum TM^n^	19	62.8 ± 14.6	31 (24–64)	ER	VAS, ASES score
Styron (2016)	IV	Tantalum TM^n^	66	66.2 (31–88)	50.2	FE, IR, ER	N
Sandow (2016)	IV	Tantalum TM^n^	10	(60–79)	24	FE	VAS, Oxford score, ASES score
Panti (2016)	IV	Tantalum TM^n^	76	69.6 (52–81)	43.2 (24–72)	FE, ABD, ER	VAS, ASES score
Endrizzi (2016)	IV	Tantalum TM^n^	73	67.5 ± 8.6 (46–85)	50.8 (24–68)	N	VAS, ASES score
Merolla (2016)	IV	Tantalum TM^n^	40	63.8 (40–75)	38 (24–42)	FE, ABD, ER	Health state, Constant score, ASES score
Gurin (2017)	IV	Tantalum TM^n^	80	N	100	N	VAS
Watson (2018)	IV	Tantalum TM^n^	36	66.36 (50–85)	34.1 (23–61)	FE, ER	VAS, SANE score, Penn score, ASES score
Common outcome measurements	Radiolucency, loosening, complication, and revision surgery (failure)						

*N* not recorded, *y* year, *m* month, *FU* follow-up, *FE* forward elevation, *ABD* abduction, *IR* internal rotation, *ER* external rotation, *VAS* visual analogue scale, *ASES* American shoulder and elbow surgeons, *SST* simple shoulder test, *SF*-12 short form-12, *UCLA* University of California at Los Angeles, *SSV* subjective shoulder value, *SANE* single alpha-numeric evaluation

^a^Unconstrained, cemented, third-generation implant (Aequalis Primary Shoulder Prosthesis; Tornier Inc., Edina, Minnesota, USA) or Aequalis prosthesis (Tornier, Mont Bonnot, France)

^b^Cofield 2 keeled all-polyethylene cemented components with a posterior augmentation (Smith and Nephew, Inc., Memphis, TN, USA)

^c^Neer II all-polyethylene components (3 M, St. Paul, MN; Kirschner Medical Corporation, Fairlawn, NJ; Biomet, Warsaw, IN, USA), Cofield 1 all-polyethylene component, Cofield 2 all-polyethylene keeled, and Cofield 2 all-polyethylene pegged components (Smith & Nephew, Memphis, TN, USA)

^d^Depuy Global Advantage with an Anchor Peg glenoid (Depuy, Warsaw, IN, USA)

^e^Affinis shoulder prosthesis (Mathys Ltd. Bettlach, Switzerland)

^f^BioModular Total Shoulder System with an all-polyethylene, cemented, pegged glenoid (Biomet, Inc., Warsaw, IN, USA)

^g^Equinoxe (Exactech, Inc., Gainesville, FL, USA)

^h^Affiniti CortiLoc glenoid (Tornier, Inc., Edina, MN, USA)

^i^Cofield II all-polyethylene pegged component (Smith & Nephew, Memphis, TN, USA)

j Cemented keeled glenoid with different backside radiuses of curvature (Tornier/Wright Medical, Memphis, TN, USA)

^k^ReUnion (Stryker, Mahwah, NJ, USA)

^l^Second generation SMR System (Lima Corporate, Villanova, Italy)

^m^Titanium metal-backed glenoid component (Sulmesh; Zimmer, Winterthur, Switzerland)

^n^Second-generation porous tantalum trabecular metal glenoid (Zimmer, Warsaw, IN, USA)

**Table 2:** Clinical outcomes of individual studies

Authors	Cases	Gain of FE (°)	Gain of ER (°)	Radiolucency	Loosening	Revision surgeries	Other reoperations
Cemented all-polyethylene glenoid components (PEG)							
Raiss (2008)	21	50.7	28.1	10 (48%)	10	0	0
Rice (2008)	14	N	21	1 (7%)	1	0	0
Fox (2009)	972	N	N	N	15	26	0
Edwards (2010)	47	N	N	N	0	2	0
Throckmorton (2010)	100	48.3	28.6	N	10	0	0
Arnold (2011)	35	N	N	5	0	0	0
Collin (2011)	56	N	N	N	20	3	2: RC repair
Walch (2011)	333	51.7	26.3	96	57	5	3: open contracture release
Young (2011)	226	39.7	23.3	144	99	37	2: Periprosthetic fracture 3: instability 2: RC repair 2: infection 2: stiffness
Raiss (2012)	39	49	24	N	N	1	0
Wirth (2012)	44	141.9	34.6	N	N	1	0
Denard (2013)	50	31	21	30 of 48	21 of 48	17	0
Greiner (2013)	97	59.6	N	9	3	7	0
Raiss (2014)	250	46.9	25.0	N	100	22	0
Gazielly (2015)	39	42.4	25.7	8	6	1	0
Gulotta (2015)	40	N	N	N	0	0	1 infection 1 biceps tendinitis
Noyes (2015)	42	30	7	8	N	1	0
Wright (2015)	24	44.2	24.8	5 of 15	0	0	0
Parks (2016)	76	31	13	14	1	7 of 80	N
Wijeratna (2016)	83	N	N	5	1	1	3 contracture release 1: RC repair 1: capsular plication
McLendon (2017)	287	N	N	N	120	36	0
Service (2017)	71	N	N	19	1	3	0
Gauci (2018)	46	40	26	N	10	10	N
Raiss (2018)	118	N	N	N	0	2	0
Sanchez-Sotelo (2018)	202	N	N	0	0	7	2
Modern design of metal-backed glenoid component (MBG)							
Castagna (2010)	35	N	N	8 (22%)	0	0	0
Fucentese (2010)	22	N	N	N	3 (14%)	3 (14%)	0
Budge (2013)	19	N	44	7 (37%)	4 (21%)	3 (16%)	N
Styron (2016)	66	70	36	N	13 of 58 (23%)	1 (2%)	N
Sandow (2016)	10	N	N	0	0	0	0
Panti (2016)	76	54.4	40.8	5 (7%)	0	0	1: RC repair
Endrizzi (2016)	73	N	N	24 of 66 (36.4%)	1 of 66 (1.5%)	1 (1%)	0
Merolla (2016)	40	N	N	2 (5%)	0	0	0
Gurin (2017)	80	N	N	N	0	2 (3%)	0
Watson (2018)	36	N	N	1 (2.8%)	1 (2.8%)	1 (3%)	N

*N* not recorded, *FE* forward elevation, *ER* external rotation, *RC* rotator cuff

### Clinical outcomes and complications of cemented PEG and modern MBG groups

**Fig. 6:**
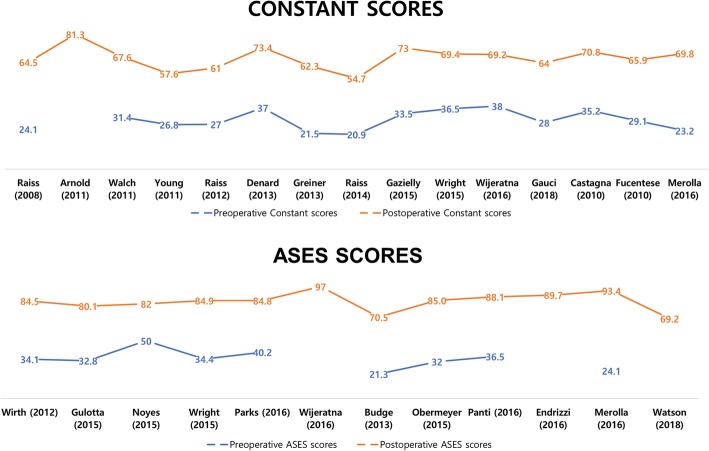
Graph showing the distribution of preoperative and postoperative clinical scores for each article. ASES, American shoulder and elbow surgeons

**Fig. 7:**
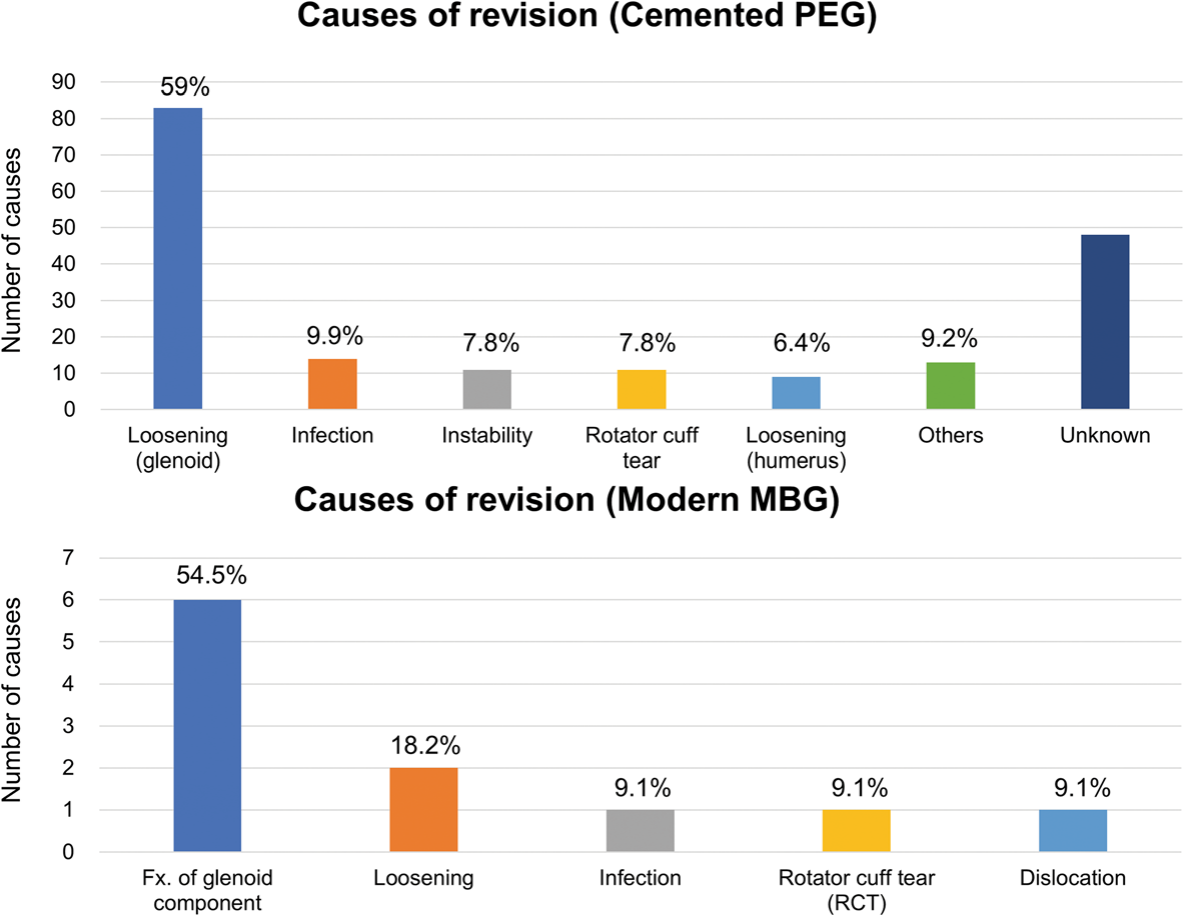
Graph showing the causes of revisions. PEG, polyethylene glenoid; MBG, metal-backed glenoid; Fx., fracture

Based on the data obtained in Table 2, an overall comparison between the two groups was performed (Table 3). The mean gain of the arc of flexion-extension (F-E) was 48.6° and 61.7° and the ER increase was 24.2° and 39.2°, the mean Constant score increase was 34.8 and 40.4, and the ASES score was 44.5 and 56.5 for cemented PEG and modern MBG, respectively (Fig. 6). Rates of radiolucent lines, loosening, and revision surgery were lower in the modern MBG group, although incomplete results did not resolve heterogeneity. The causes of the revision are summarized in Fig. 7; the most common cause of reoperation for the cemented PEG group was loosening of glenoids (83 out of 141 known causes, 59.0%), and fractures of glenoid components for the modern MBG group (6 out of 11 known causes, 54.5%).

**Table 3:** Summary of cemented PEG and modern MBG

		Cemented PEG (*n* = 3312)	Modern MBG (*n* = 457)	*P*–value
Age (years)	Number of cases/articles	3062/24	367/8	NA
	**Mean**	**66.4 (21–93)**	**66.5 (31–88)**	
Follow–up duration (months)	Number of cases/articles	3312/25	457/10	NA
	**Mean**	**73.1 (12–211)**	**56.1**	
Gain of FE (°)	Number of cases/articles	1387/14	142/2	NA
	**Mean**	**48.6**	**61.7**	
Gain of ER (°)	Number of cases/articles	1304/14	161/3	NA
	**Mean**	**24.2**	**39.2**	
Constant score improvement	Number of cases/articles	1208/9	3/97	NA
	**Mean**	**34.8**	**40.4**	
ASES score improvement	Number of cases/articles	226/5	135/3	NA
	**Mean**	**44.5**	**56.5**	
Primary osteoarthritis	**Yes (%)**	**2866 (86.5%)**	**350 (88.4%)**	0.310
	No (%)	446 (13.5%)	46 (11.6%)	
	Diagnosis unknown	1	61	
Radiolucent lines	**Present (%)**	**354 (27.2%)**	**22 (4.9%)**	NA
	Absent (%)	948 (72.8%)	427 (95.1%)	
	Not reported	2010	8	
Loosening	**Present (%)**	**465 (14.6%)**	**22 (4.9%)**	NA
	Absent (%)	2720 (85.4%)	427 (95.1%)	
	Not reported	127	8	
Revision surgery	**Present (%)**	**189 (5.7%)**	**11 (2.4%)**	NA
	Absent (%)	3127 (94.3%)	446 (97.6%)	
	Not reported	0	0	

*PEG* all-polyethylene glenoid, *MBG* metal-backed glenoid, *NA* not applicable, *FE* forward elevation, *ER* external rotation, *ASES* American shoulder and elbow surgeons

### Scatter plots and subgroup analysis according to the FU duration

**Fig. 8:**
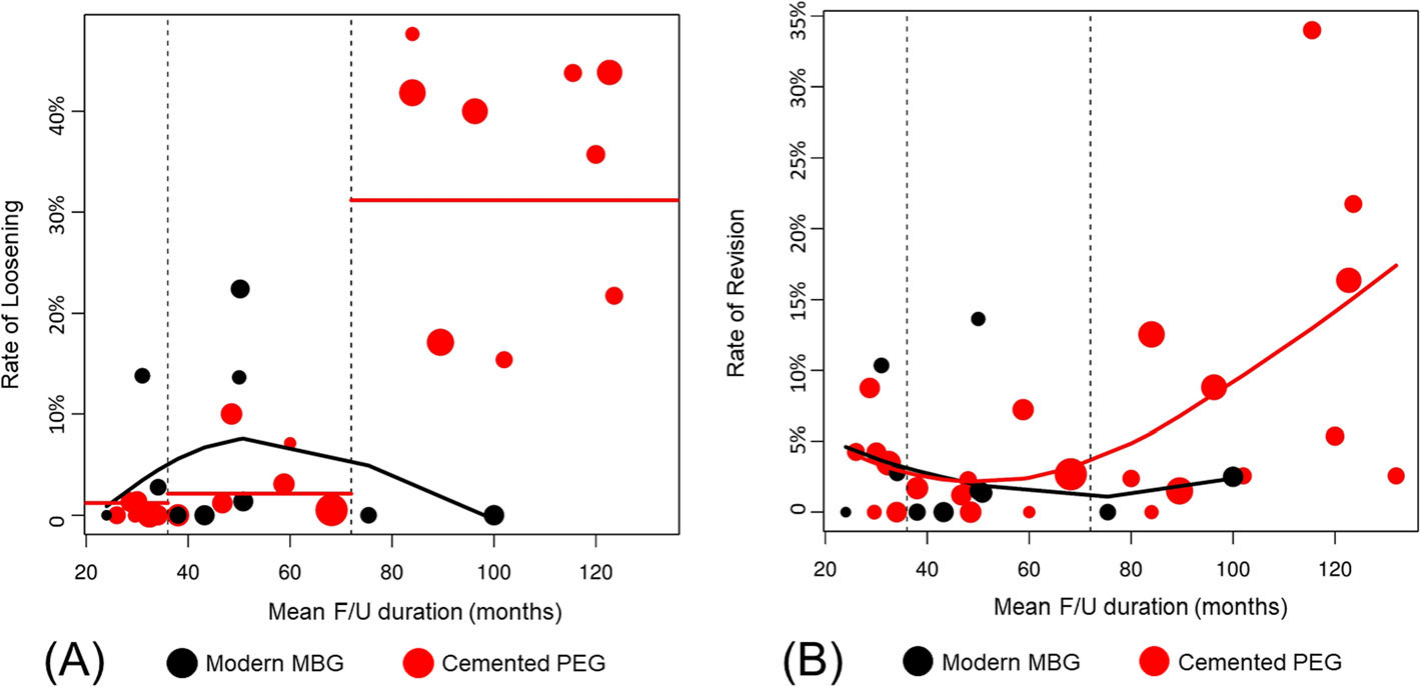
(**a**) Scatter plots showing the loosening rates for each study. **b** Scatter plots showing the revision rates for each study. PEG, polyethylene glenoid; MBG, metal-backed glenoid; Vertical dotted lines, thresholds (3 and 6 years) for dividing < 36-month, 36–72-month, and > 72-month subgroups; Black line, the trendline of modern metal-backed glenoid group; Red line, trendline of cemented polyethylene glenoid group

**Fig. 9:**
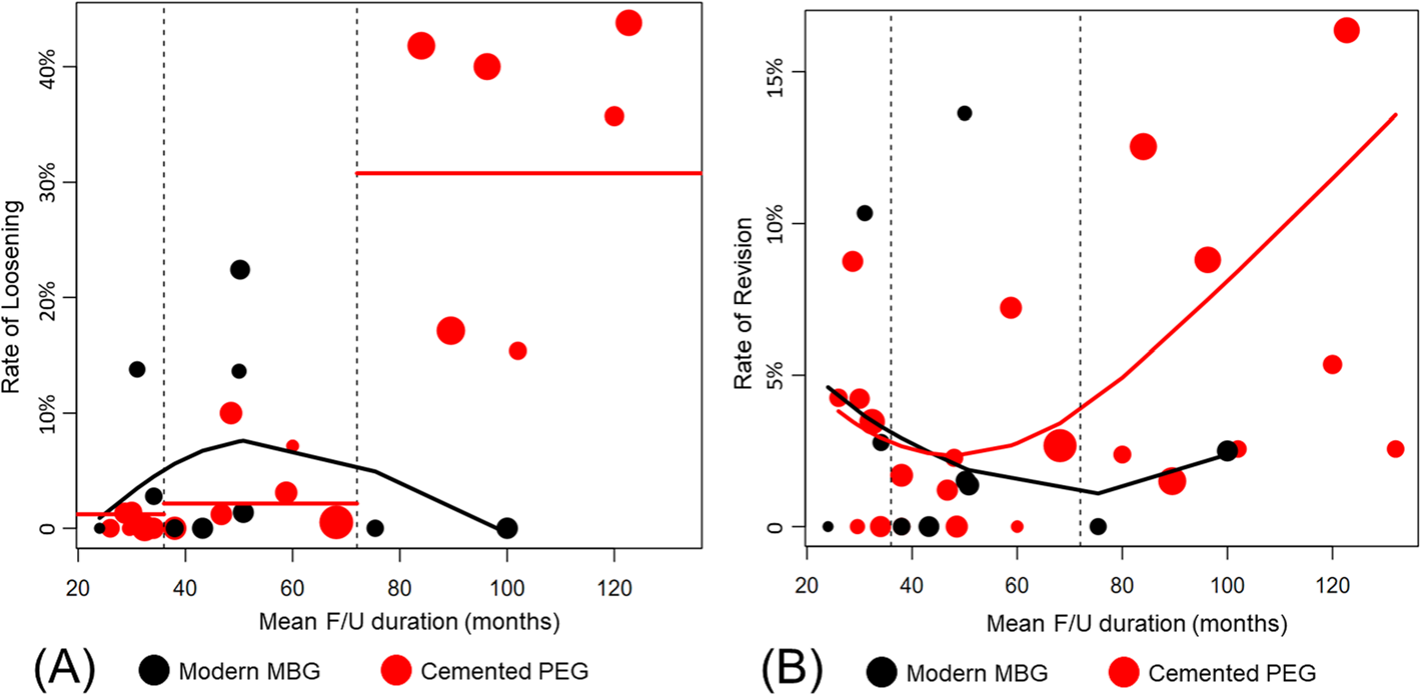
(**a**) Scatter plots showing the loosening rates for each study excluding three studies which included only young adults (**b**) Scatter plots showing the revision rates for each study excluding three studies which included only young adults. PEG, polyethylene glenoid; MBG, metal-backed glenoid; Vertical dotted lines, thresholds (3 and 6 years) for dividing <36-month, 36–72-month, and > 72-month subgroups; Black line, trendline of modern metal-backed glenoid group; Red line, trendline of cemented polyethylene glenoid group

We performed additional scatter plot and subgroup analyses according to the FU duration, which showed a heterogeneous pattern. The trend lines showed that the MBG group tended to have lower loosening and revision rates than the PEG group over time (Fig. 8a and b). Table 4 shows the results of subgroup analysis according to the FU period. The results of < 36-month and 36–72-month subgroups showed that cemented PEG showed good results in terms of radiolucency and loosening, but that there was no significant difference in failure rate (*P* = 0.754 and 0.829 for < 36-month and 36–72-month subgroups). In contrast, in > 72-month subgroup, modern MBG showed better results in terms of loosening (*P* < 0.001) and revision rates (*P* = 0.006). We additionally compared two groups, after excluding three studies which included only young adults [14, 17, 24]. The scatter plot analysis and subgroup analysis according to the FU duration showed the same trend as that of the main analysis (Table 5, Fig. 9a and Fig. 9b).

**Table 4:** Subgroup analysis according to the follow-up duration

Items	<36-month subgroup^a^		36–72-month subgroup^b^		>72-month subgroup^c^	
	PEG	MBG	PEG	MBG	PEG	MBG
Age (years)	66.7	64.8	66.9	67.3	65.3	62.7
Number of radiolucency (%)	38/439 (7.3%)	8/65 (12.3%)	20/229 (8.7%)	31/182 (17.0%)	296/709 (41.7%)	8/35 (22.9%)
	*P* = 0.355		***P*** = 0.015 *		***P*** = 0.033 *	
Number of loosening	2/420 (0.5%)	5/65 (7.7%)	19/1459 (1.3%)	17/269 (6.3%)	443/1306 (33.9%)	0/115 (0%)
	***P*** < 0.001 *		***P*** < 0.001 *		***P*** < 0.001 *	
Number of failure (=revision)	19/424 (4.5%)	4/65 (6.2%)	37/1503 (2.5%)	5/277 (1.8%)	133/1389 (9.6%)	2/115 (1.7%)
	*P* = 0.754		*P* = 0.829		***P*** = 0.002 *	

*FU* follow-up, *PEG* cemented all-polyethylene glenoid, *MBG* metal-backed glenoid

^a^follow-up duration less than 36 months

^b^follow-up duration between 36 and 72 months

^c^follow-up duration more than 72 months

*statistically significant change

**Table 5:** Subgroup analysis except for 3 articles which included only young adults

Items	<36-month subgroup^a^		36–72-month subgroup^b^		> 72-month subgroup^c^	
	PEG	MBG	PEG	MBG	PEG	MBG
Age (years)	66.7	64.8	66.9	67.3	66.7	62.7
Number of radiolucency (%)	38/439 (7.3%)	8/65 (12.3%)	20/229 (8.7%)	31/182 (17.0%)	256/640 (40.0%)	8/35 (22.9%)
	*P* = 0.355		***P*** = 0.015 *		***P*** **= 0.05**	
Number of loosening	2/420 (0.5%)	5/65 (7.7%)	19/1459 (1.3%)	17/269 (6.3%)	402/1191 (33.8%)	0/115 (0%)
	***P*** < 0.001 *		***P*** < 0.001 *		***P*** < 0.001 *	
Number of failure (=revision)	19/424 (4.5%)	4/65 (6.2%)	37/1503 (2.5%)	5/277 (1.8%)	106/1272 (8.3%)	2/115 (1.7%)
	*P* = 0.754		*P* = 0.829		***P*** = 0.006 *	

*FU* follow-up, *PEG* cemented all-polyethylene glenoid, *MBG* metal-backed glenoid

^a^follow-up duration less than 36 months

^b^follow-up duration between 36 and 72 months

^c^follow-up duration more than 72 months

*statistically significant change

## Discussion

Although failure rates did not differ significantly between the two glenoid types in < 36-month and 36–72-month subgroups, modern MBGs were found to have lower radiolucency, loosening, and failure rates than cemented PEG in > 72-month subgroup (*P* = 0.033, < 0.001, and 0.006, respectively). This is in line with the results obtained from the scatter plot analysis. Also, the gains of FE and ER, Constant score, and ASES score of the modern MBG group were not lower than those of cemented PEG. Taken together, these results show that the modern MBG is comparable to the cemented PEG, with promisingly better results in a few of these aspects.

The trends in outcomes were found to differ between the two groups as the FU duration increased. In the cemented PEG group, loosening and failure rate typically increased as the FU duration increased. In contrast, > 72-month subgroup was comparable to < 36-month subgroup in the modern MBG group. This may be because it is possible that the MBG was stably fixed, and that bony ingrowth was sufficient. If the modern MBG design caused stable fixation and bony ingrowth as the design originally intended, it makes sense that there were some initial failures in the modern MBG group and that the results of > 72-month subgroup of the modern MBG were better than PEG. Moreover, it is possible that the error occurred due to the lack of studies with a long-term FU on modern MBGs. In order to confirm this conclusion, more long-term FU studies on modern MBGs should be performed.

A previous systematic review by Papadonikolakis and Matsen compared rates of complications and revision surgeries between MBG and PEG. They included all designs of MBGs up to 2013 in the same group and reported that MBGs showed significantly higher revision rates than PEG [3]. Categorical data such as loosening and revision were analyzed by crosstab analysis as in this study. The review is a well-performed study that has served as a reference for the selection of glenoid components. We tried to increase the credibility of the analytical results by conducting heterogeneity assessments and adjustments that did not appear to be performed in the previous review.

The MBG was designed to induce bone ingrowth using the porous-coated component on the glenoid contact surface, and smooth ROM on the joint surface using the PE component. Because of this, they were expected to be the ideal component. However, the results of clinical studies using the conventional MBG design were disappointing [2, 16, 17, 44–51]. These failures were caused by several factors. First, MBG failure is often associated with PE wear, which is often caused by thinner PE thickness in these designs due to the metal back [16]. Second, overstuffing of joints can be induced to ensure sufficient PE thickness, resulting in loosening and rotator cuff tears, which ultimately leads to joint instability. Third, breakages of rods and screws may occur that are not caused by cemented PEG.

Attempts have been made to improve this by modifications to the MBG’s design. Second-Generation SMR MBG (SMR System, Lima Corporate, Villanova, di San Daniele, Udine, Italy) is the representative of this modern design. Castagna and Garofalo reported good results using this instrument, due to the curved-backed and less conforming shape of the glenoid, the stiff and thick metal back (5 mm) for reducing stress and wear on the PE component, applying hydroxyapatite to pegs as well as the baseplate, and initial strong fixation using two screws and one central peg [4]. Other representative modern designs are Zimmer’s trabecular porous tantalum and titanium TM. The first generation of TM, the Sulmesh, consists of several titanium meshes, with four pegs protecting the metal back. The second-generation TM shows an improved design with porous tantalum keels. Recently, many clinical studies on these modern designs of MBG have been reported, and with the exception of a few studies, good results were reported with low failure rates and fast bony ingrowth around keels [4–6, 37–43, 52].

A study by Page et al. on similar topics analyzed glenoid revision rates using the Australian Orthopaedic Association National Joint Replacement Registry, which began in 2004. Cementless MBG was classified into modular type and fixed type in the study. Among them, SMR L2 and TM glenoid which were considered as modern design in this review were included in the analysis. Cementless glenoids showed a significantly higher revision rate than cemented glenoids [53]. Contrary to the results presented by Castagna et al. [4], The SMR L2 design showed higher revision rate than other designs. Based on the result, SMR L2 was withdrawn from the market in Austrailia. TM glenoids, on the other hand, showed the same low revision rate in this review and the study by Page et al. [53]. Results of this review and the study by Page et al. suggest that surgeons should be cautious in MBG selection because it can produce different results for different designs. Among MBG designs, TM glenoids are most promising and comparable to cemented glenoids.

This study has several limitations. First, there is no clear global consensus on the distinction between modern design and conventional design. However, a rationale was found through a review article on glenoid components by Castagna and Garofalo [10] and three models were defined as modern designs. Second, there is the possibility of remaining heterogeneity between studies. We thoroughly discussed this point at the research design stage and conducted data analysis and pooling after sufficient distribution analysis and adjustment of bias. Third, studies use different criteria for the definition of radiolucency and loosening in the main outcomes. Here, these were summarized using the most common and objective items as was possible, and credibility was increased by eliminating ambiguous data. Ultimately, the most objective and ultimate outcome indicator is failure or revision rate, and the failure rate results presented in this review suggest that modern MBG is promising. Fourth, cemented PEGs were not divided into conventional and modern designs. Cemented PEGs have long been the standard glenoid designs for TSA, showing relatively consistent results and trends. Thus, we could not clearly classify them into modern and conventional designs.

The fifth limitation is the lack of longer-term FU data across all implants. It is especially true in the modern MBG group, leading to the shortened criteria for dividing subgroups (36 and 72 months). This is because many surgeons still prefer cemented PEGs, and the modern designs of MBGs are still quite new. Since new designs are being developed in addition to the modern MBGs included in this study, a definite consensus on MBGs may be formed if more long-term FU studies on the MBGs as well as cemented PEGs are actively conducted.

## Conclusion

The modern MBG component, especially TM glenoid, seems to be a promising alternative to cemented PEGs, based on subgroup revision rates according to the follow-up duration and overall results of ROM and clinical scores. All polyethylene glenoids tend to increase loosening and failure over time. The modern MBG seems to have no difference in failure, at least in the < 36-month and 36–72-month subgroups compared to the cemented PEG. More long-term follow-up studies on modern MBG should be ultimately conducted.

## Data Availability

Not applicable.

## Abbreviations

ASES: American shoulder and elbow surgeons
ER: external rotation
F-E: flexion-extension
FE: forward elevation
FU: follow up
MBG: metal-backed glenoid
MINORS: methodological index for non-randomized studies
N: not recorded
PE: polyethylene
PEG: cemented polyethylene glenoid
RC: rotator cuff
ROM: range of motion
TM: trabecular metal
TSA: total shoulder arthroplasty
VAS: visual analogue scale

## References

[1] Oh JH, Song BW (2011). The current state of Total shoulder Arthroplasty. Clin Shoulder Elbow.

[2] Boileau P, Avidor C, Krishnan SG, Walch G, Kempf JF, Mole D (2002). Cemented polyethylene versus uncemented metal-backed glenoid components in total shoulder arthroplasty: a prospective, double-blind, randomized study. J Shoulder Elb Surg.

[3] Papadonikolakis A, Matsen FA (2014). Metal-backed Glenoid components have a higher rate of failure and fail by different modes in comparison with all-polyethylene components: a systematic review. J Bone Joint Surg Am.

[4] Castagna A, Randelli M, Garofalo R, Maradei L, Giardella A, Borroni M (2010). Mid-term results of a metal-backed glenoid component in total shoulder replacement. The Journal of bone and joint surgery British volume.

[5] Fucentese SF, Costouros JG, Kuhnel SP, Gerber C (2010). Total shoulder arthroplasty with an uncemented soft-metal-backed glenoid component. J Shoulder Elb Surg.

[6] Merolla G, Chin P, Sasyniuk TM, Paladini P, Porcellini G (2016). Total shoulder arthroplasty with a secondgeneration tantalum trabecular metal-backed glenoid component. Bone and Joint Journal.

[7] Moher D, Liberati A, Tetzlaff J, Altman DG, Group P (2009). Preferred reporting items for systematic reviews and meta-analyses: the PRISMA statement. PLoS Med.

[8] The Oxford 2011 Levels of Evidence [http://www.cebm.net/index.aspx?o=5653].

[9] Slim K, Nini E, Forestier D, Kwiatkowski F, Panis Y, Chipponi J (2003). Methodological index for non-randomized studies (MINORS): development and validation of a new instrument. ANZ J Surg.

[10] Castagna A, Garofalo R (2019). Journey of the glenoid in anatomic total shoulder replacement. Should Elb.

[11] Molé D, Roche O, Riand N, Lévigne C, Walch G, BP WG (1999). Cemented glenoid component: results in osteoarthritis and rheumatoid arthritis. *Shoulder arthroplasty*.

[12] Arnold RM, High RR, Grosshans KT, Walker CW, Fehringer EV (2011). Bone presence between the central peg's radial fins of a partially cemented pegged all poly glenoid component suggest few radiolucencies. J Shoulder Elb Surg.

[13] Collin P, Tay AK, Melis B, Boileau P, Walch G (2011). A ten-year radiologic comparison of two-all polyethylene glenoid component designs: a prospective trial. J Shoulder Elb Surg.

[14] Denard PJ, Raiss P, Sowa B, Walch G (2013). Mid- to long-term follow-up of total shoulder arthroplasty using a keeled glenoid in young adults with primary glenohumeral arthritis. J Shoulder Elb Surg.

[15] Edwards TB, Labriola JE, Stanley RJ, O'Connor DP, Elkousy HA, Gartsman GM (2010). Radiographic comparison of pegged and keeled glenoid components using modern cementing techniques: a prospective randomized study. J Shoulder Elb Surg.

[16] Fox TJ, Cil A, Sperling JW, Sanchez-Sotelo J, Schleck CD, Cofield RH (2009). Survival of the glenoid component in shoulder arthroplasty. J Shoulder Elb Surg.

[17] Gauci MO, Bonnevialle N, Moineau G, Baba M, Walch G, Boileau P (2018). Anatomical total shoulder arthroplasty in young patients with osteoarthritis: all-polyethylene versus metal-backed glenoid. The bone & joint journal.

[18] Gazielly DF, Scarlat MM, Verborgt O (2015). Long-term survival of the glenoid components in total shoulder replacement for arthritis. Int Orthop.

[19] Greiner S, Berth A, Kaab M, Irlenbusch U (2013). Glenoid morphology affects the incidence of radiolucent lines around cemented pegged polyethylene glenoid components. Arch Orthop Trauma Surg.

[20] Gulotta LV, Chambers KL, Warren RF, Dines DM, Craig EV (2015). No differences in early results of a hybrid glenoid compared with a pegged implant. Clin Orthop Relat Res.

[21] McLendon PB, Schoch BS, Sperling JW, Sanchez-Sotelo J, Schleck CD, Cofield RH (2017). Survival of the pegged glenoid component in shoulder arthroplasty: part II. J Shoulder Elb Surg.

[22] Noyes MP, Meccia B, Spencer EE (2015). Five- to ten-year follow-up with a partially cemented all-polyethylene bone-ingrowth glenoid component. J Shoulder Elb Surg.

[23] Parks DL, Casagrande DJ, Schrumpf MA, Harmsen SM, Norris TR, Kelly JD (2016). Radiographic and clinical outcomes of total shoulder arthroplasty with an all-polyethylene pegged bone ingrowth glenoid component: prospective short- to medium-term follow-up. J Shoulder Elb Surg.

[24] Raiss P, Aldinger PR, Kasten P, Rickert M, Loew M (2008). Total shoulder replacement in young and middle-aged patients with glenohumeral osteoarthritis. The Journal of bone and joint surgery British volume.

[25] Raiss P, Edwards TB, Deutsch A, Shah A, Bruckner T, Loew M (2014). Radiographic changes around humeral components in shoulder arthroplasty. J Bone Joint Surg Am.

[26] Raiss P, Godenèche A, Wittmann T, Schnetzke M, Bruckner T, Neyton L (2018). Short-term radiographic results of a cemented polyethylene keeled glenoid component with varying backside radiuses of curvature. J Shoulder Elb Surg.

[27] Raiss P, Schmitt M, Bruckner T, Kasten P, Pape G, Loew M (2012). Results of cemented total shoulder replacement with a minimum follow-up of ten years. J Bone Joint Surg Am.

[28] Rice RS, Sperling JW, Miletti J, Schleck C, Cofield RH (2008). Augmented glenoid component for bone deficiency in shoulder arthroplasty. Clin Orthop Relat Res.

[29] Sanchez-Sotelo J, Nguyen NTV, Morrey M (2018). Anatomic Total shoulder Arthroplasty using a self-pressurizing pegged bone-preserving cemented Glenoid component: a 2- to 5-year follow-up study. Journal of Shoulder and Elbow Arthroplasty.

[30] Hsu JE, Somerson JS, Russ SM, Matsen FA, Service BC (2017). Does postoperative Glenoid retroversion affect the 2-year clinical and radiographic outcomes for Total shoulder Arthroplasty?. Clin Orthop Relat Res.

[31] Throckmorton TW, Zarkadas PC, Sperling JW, Cofield RH (2010). Pegged versus keeled glenoid components in total shoulder arthroplasty. J Shoulder Elb Surg.

[32] Walch G, Young AA, Melis B, Gazielly D, Loew M, Boileau P (2011). Results of a convex-back cemented keeled glenoid component in primary osteoarthritis: multicenter study with a follow-up greater than 5 years. J Shoulder Elb Surg.

[33] Wijeratna M, Taylor D, Lee S, Hoy G, Evans MC (2016). Clinical and radiographic results of an all-polyethylene pegged bone-ingrowth glenoid component. J Bone Joint Surg (Am Vol).

[34] Wirth MA, Loredo R, Garcia G, Rockwood CA, Southworth C, Iannotti JP (2012). Total shoulder arthroplasty with an all-polyethylene pegged bone-ingrowth glenoid component: a clinical and radiographic outcome study. J Bone Joint Surg Am.

[35] Wright TW, Grey SG, Roche CP, Wright L, Flurin PH, Zuckerman JD (2015). Preliminary Results of a Posterior Augmented Glenoid Compared to an all Polyethylene Standard Glenoid in Anatomic Total Shoulder Arthroplasty. Bulletin of the Hospital for Joint Disease (2013).

[36] Young A, Walch G, Boileau P, Favard L, Gohlke F, Loew M (2011). A multicentre study of the long-term results of using a flat-back polyethylene glenoid component in shoulder replacement for primary osteoarthritis. The Journal of bone and joint surgery British volume.

[37] Budge MD, Nolan EM, Heisey MH, Baker K, Wiater JM (2013). Results of total shoulder arthroplasty with a monoblock porous tantalum glenoid component: a prospective minimum 2-year follow-up study. J Shoulder Elb Surg.

[38] Endrizzi DP, MacKenzie JA, Henry PDG (2016). Early debris formation with a porous tantalum glenoid component radiographic analysis with 2-year minimum follow-up. J Bone Joint Surg (Am Vol).

[39] Panti JP, Tan S, Kuo W, Fung S, Walker K, Duff J (2016). Clinical and radiologic outcomes of the second-generation trabecular metal glenoid for total shoulder replacements after 2-6 years follow-up. Arch Orthop Trauma Surg.

[40] Sandow M, Schutz C (2016). Total shoulder arthroplasty using trabecular metal augments to address glenoid retroversion: the preliminary result of 10 patients with minimum 2-year follow-up. J Shoulder Elb Surg.

[41] Styron JF, Marinello PG, Peers S, Seitz WH (2016). Survivorship of trabecular metal anchored Glenoid Total shoulder Arthroplasties. Tech Hand Upper Extrem Surg.

[42] Gurin D, Seitz WH (2017). The emerging role of the non-cemented glenoid in total shoulder arthroplasty. Semin Arthroplast.

[43] Watson ST, Gudger GK, Long CD, Tokish JM, Tolan SJ (2018). Outcomes of trabecular metal-backed glenoid components in anatomic total shoulder arthroplasty. J Shoulder Elb Surg.

[44] Vuillermin CB, Trump ME, Barwood SA, Hoy GA (2015). Catastrophic failure of a low profile metal-backed glenoid component after total shoulder arthroplasty. International journal of shoulder surgery.

[45] Martin SD, Zurakowski D, Thornhill TS (2005). Uncemented glenoid component in total shoulder arthroplasty. Survivorship and outcomes. J Bone Joint Surg Am.

[46] Montoya F, Magosch P, Scheiderer B, Lichtenberg S, Melean P, Habermeyer P (2013). Midterm results of a total shoulder prosthesis fixed with a cementless glenoid component. J Shoulder Elb Surg.

[47] Rosenberg N, Neumann L, Modi A, Mersich IJ, Wallace AW (2007). Improvements in survival of the uncemented Nottingham Total shoulder prosthesis: a prospective comparative study. BMC Musculoskelet Disord.

[48] Tammachote N, Sperling JW, Vathana T, Cofield RH, Harmsen WS, Schleck CD (2009). Long-term results of cemented metal-backed glenoid components for osteoarthritis of the shoulder. J Bone Joint Surg Am.

[49] Taunton MJ, McIntosh AL, Sperling JW, Cofield RH (2008). Total shoulder arthroplasty with a metal-backed, bone-ingrowth glenoid component. Medium to long-term results. J Bone Joint Surg Am.

[50] Wallace AL, Phillips RL, MacDougal GA, Walsh WR, Sonnabend DH (1999). Resurfacing of the glenoid in total shoulder arthroplasty. A comparison, at a mean of five years, of prostheses inserted with and without cement. J Bone Joint Surg Am.

[51] Katz D, Kany J, Valenti P, Sauzieres P, Gleyze P, El Kholti K (2013). New design of a cementless glenoid component in unconstrained shoulder arthroplasty: a prospective medium-term analysis of 143 cases. European journal of orthopaedic surgery & traumatology : orthopedie traumatologie.

[52] Obermeyer T, Cagle PJ, Parsons BO, Flatow EL (2015). Midterm Follow-Up of Metal-Backed Glenoid Components in Anatomical Total Shoulder Arthroplasties. American journal of orthopedics (Belle Mead, NJ).

[53] Page RS, Pai V, Eng K, Bain G, Graves S, Lorimer M (2018). Cementless versus cemented glenoid components in conventional total shoulder joint arthroplasty: analysis from the Australian Orthopaedic Association national joint replacement registry. J Shoulder Elb Surg.

